# Internal and external drivers for compliance with the COVID-19 preventive measures in Slovenia: The view from general deterrence and protection motivation

**DOI:** 10.1371/journal.pone.0259675

**Published:** 2021-11-15

**Authors:** Anže Mihelič, Luka Jelovčan, Kaja Prislan

**Affiliations:** Faculty of Criminal Justice and Security, University of Maribor, Ljubljana, Slovenia; Bucharest University of Economic Studies, ROMANIA

## Abstract

The emergence of a pandemic is usually accompanied by different measures–economic, social, preventive, and (self)protective. In the case of the COVID-19, several preventive measures were formally enforced by state authorities in the majority of countries worldwide. Thus, during the COVID-19 pandemic, the intertwining of formal and informal social control could be observed. Hence, in this study a cross-sectional design was chosen to explore the issue in Slovenia. To the best of our knowledge, this research is the first in the current literature to empirically test the general deterrence theory in pandemic circumstances (as external factors predicting individuals’ compliance with the COVID-19 preventive measures). The results suggest an important role of informal punishment, with perceived informal severity being the only statistically significant factor from the general deterrence theory. In contrast to external factors, internal factors play a significantly greater role in promoting people’s self-protective behavior in pandemic circumstances. During the unknown, the uncertain and delicate situations with which people have no previous experience, both personal beliefs about the effectiveness of measures and perceived self-efficacy are more important than fear of formal sanctions.

## 1 Introduction

Enforcing mandatory measures that restrict fundamental rights and freedoms and punishing the violators of these measures are common for countries declaring a state of emergency. Such measures are especially prevalent in states enforcing a "war on crime" [1] or are faced with emergencies related to terrorist attacks [2]. Less commonly, states also restrict certain fundamental human rights and freedoms to protect public health. An example of such restrictions can be found in British prisons, where authorities restricted certain rights to stop spreading HIV [3]. Similarly, in 2009, governments worldwide restricted certain fundamental human rights and freedoms to stop spreading the A/H1N1 virus [4]. Preventive protective measures (e.g., isolation zones, mandatory use of personal protection equipment, controlling travel) also accompanied the Ebola epidemic in 2014 [5].

To stop the spreading of the SARS-CoV-2 virus states worldwide issued a variety of measures to protect public health [6, 7]. Among the measures were also formally issued regulations, where COVID-19 preventive behavior is required by law. Since violations of the regulations meant violation of law, penalties were also often issued. Along with formal responses to violating mandatory COVID-19 preventive measures, violations were often accompanied by informal social responses. In such emotionally charged situations, public shaming is common in various media, as people seek justice, consolation, or even entertainment [8]. Several studies have already reported cases of public shaming, discrimination, and polarization of citizens in times of the COVID-19 pandemic [9, 10]. Such informal social control also contributed to formal responses. For example, Slovenian authorities issued several fines based on other citizens’ reports or even posts on social networks [11]. However, the effects of protective measures were diverse. While the measures contributed to the overall containment of the disease, they also had negative consequences. For example, a study conducted during the COVID-19 pandemic in China indicates that the danger of the pandemic to the mental health of the citizens should not be overlooked [12].

The researchers have already studied protective behavior in times of pandemics or epidemics. To explain the intentions to self-protect, several established theories and models were used, such as the Health belief model [13], the Protection motivation theory [14], and the Theory of planned behavior [15]. The studies mentioned above suggest that the individual’s intention to comply with mandatory protective measures during epidemics and pandemics is influenced by many factors; however, all these studies assume that the individual makes a decision based on their own beliefs [16]. These studies thus primarily focus on the importance of internal factors. However, relying solely on people’s personal willingness to take action does not guarantee that the measures will be followed [17]. Thus, external factors, which have a potential impact on individual decision-making should, also be considered.

Among factors influencing protective behavior, external cues for action have already proven to be a significant predictor of the decision to comply with recommended protective measures [18]. External cues of action refer to information that encourages the citizens to uptake protective measures. Such cues mainly derive from closer circles, such as family, friends, and official authorities, such as the state [19]. This information is primarily related to the disease and its symptoms [20]. Since several protective measures worldwide were government-enforced, it is reasonable to assume that in addition to internal factors (stemming from the health belief model, the protection motivation theory, and the theory of planned behavior), external factors, such as the threat of punishment, also influence the decision to uptake COVID-19 preventive measures. Hence, general deterrence theory, which is generally accepted in criminological research and contributes to understanding people’s willingness to (dis)respect social norms, could prove to be a significant predictor of government-enforced protective behavior. However, it has not yet been applied to the restrictive measures in a pandemic condition.

To the best of our knowledge, the role of formal and informal sanctions has not yet been researched in times of pandemic or epidemic. In this paper, we focus on internal and external factors by using the general deterrence theory and the protection motivation theory, which we assume contribute to compliance with the COVID-19 preventive measures (i.e., governmental protective instructions). Our research will answer the following research questions:

- **RQ1:** How do respondents perceive the severity and certainty of formal and informal sanctions?
- **RQ2:** How do respondents perceive their vulnerability, severity of the virus, their self-efficacy, and efficacy of the protective measures?
- **RQ3:** What was the general attitude of respondents towards governmental protective instructions?
- **RQ4:** Which internal and external factors are associated with the self-reported compliance to governmental protective instructions?

Our research will offer an insight into how general deterrence impacts the decision to uptake protective measures compared to (internal) factors deriving from the Protection motivation theory.

## 2 Theoretical background

### 2.1 Protection motivation theory

Research in the area of safety behavior and compliance tends to investigate protective behavior to explain individuals’ motivation and attitudes towards preventative measures. Different behavioral and psychological premises are used in this context, namely, the Protection-motivation theory (PMT), the Health-belief model (HBM), and the fear appeal concept, which all emphasize the importance of risk/threat and coping strategies perception on the adoption of preventative and security measures.

PMT is a cognitive model for predicting individuals’ behavior and was originally proposed by Rogers [21]. Similar to HBM (proposed by Rosenstock [22]), it has been widely adopted as a framework for investigating health-related behavior [23], for the purpose of understanding and changing an individual’s attitude towards health and responding to messages about health risks [24]. PMT follows the theoretical premises of HBM and its emphasis on the cognitive processes mediating attitudinal and behavioral change [25, 26]. The PMT explores why and how motivation and actions change when people are confronted by threats [27]. Nowadays it is commonly used not only in investigating health-related behavior, but also in exploring motivation for the use of protective (security and safety) measures in different fields (e.g., information security, workplace safety, natural hazards).

PMT assumes that two main cognitive processes affect individuals’ behavior: threat assessment and coping assessment, which consist of six components that influence the commitment or abandonment of motivation for safety behavior: (1) perceived threat severity (belief of critical consequences or extent of potential harm caused by a specific threat), (2) perceived vulnerability (probability of threat realization), (3) perceived effectiveness of preventive measures, (4) perceived self-efficacy (assessment of ability to effectively deal with the threat by evaluating preventive measures), (5) the rewards for maladaptive behavior, and (6) the cost of responding to threats [24].

The threat appraisal process is related to the individuals’ perception of threats. This means that when assessing a threat, the individual first assesses the severity of the threat and the likelihood of its realization, and then compares them with the benefits of a non-adaptive behavior. An individual can change or adapt his existing behavior, depending on the type of danger, his own perception of vulnerability, and internal and external satisfaction related to risky behavior [28]. For a behavioral change to occur, the individual must therefore be aware that he or she is exposed to risks and that benefits do not outweigh the risks of consequences if they continue with their risky behavior. When an individual notices the danger of a situation and is intimidated by the consequences, the process of finding different strategies for dealing with the threat begins. Next, the coping appraisal process includes weighing the losses and benefits of adaptive or non-adaptive behavior, in relation to the perceived effectiveness of the proposed behavior (perceived effectiveness), the perceived ability to execute the proposed behavior (perceived self-efficacy), and the estimated losses (costs of proposed behavior) [24, 26].

Studies investigating individuals’ self-protective behavior and motivation to adopt preventative measures found that perceived threat severity and vulnerability increase the intention for protective behavior, while maladaptive awards have negative influence on behavioral change [24, 29]. Furthermore, if an individual believes that a certain measure will be effective in reducing a threat and he or she is able to perform the proposed preventative activity (i.e., has appropriate skills) protective measures are more likely to be implemented [30, 31]. However, the concerns about the costs of performing protective behavior in turn negatively affect behavioral change [32].

As noted, the PMT framework is also used for investigating fear appeals [25, 26]. The authors originally assumed that provoking fear of a certain health risk and susceptibility to diseases, as well as recommendations on behavior in the manner of proposed safety measures, reduces or even eliminates the risk of infection with a certain disease [33]. Motivation for protection occurs when people are exposed to danger to their health or life. Exposure to health hazards triggers two cognitive assessment processes [34]: (1) risk assessment (a cognitive assessment that determines why and to what extent a particular situation is stressful for an individual) and (2) coping with stress (the process by which an individual regulates emotions). These processes lead to an adaptive or non-adaptive response [28]. Thus, certain studies based on PMT confirmed that perceived threat severity and vulnerability are associated with fear, which in turn impacts behavioral intention to comply with recommended safety and security measures [35, 36]. Based on these findings we propose the following hypotheses:

**H1a:** Perceived vulnerability to the virus is positively associated with the compliance to government instructions (protective behavior).
**H1b:** Perceived severity of the consequences as a result of the virus infection is positively associated with the compliance to government instructions (protective behavior).
**H1c:** Perceived self-efficacy is positively associated with the compliance to government instructions (protective behavior).
**H1d:** Perceived measure efficacy is positively associated with the compliance to government instructions (protective behavior).

### 2.2 General deterrence theory

Several countries adopted protective measures against SARS-CoV-2 to protect public health [6]. However, the voluntariness of COVID-19 preventive measures varied across countries. Sweden, for example, was known for the permissive nature of issued measures, as the protective measures were only recommended [37]. However, on the other hand, Serbia used repressive authorities to ensure the compliance of the public with issued measures [38]. The majority of European countries followed such an authoritative model and issued more restrictive approaches in various forms. A study conducted during the COVD-19 pandemic in Japan suggests that relying solely on the voluntariness of citizens to comply with protective measures is not sufficient since only a third of all citizens fully follow all recommended protective measures [17]. Along with issuing recommendations on COVID-19 preventive behavior, Slovenia declared a national epidemic and issued several legislative regulations to stop spreading the COVID-19. Movement and gathering of people in public places and crossing the municipal border were prohibited, among others. Even though protective measures are issued with the intent to maintain public health, the majority of them infringe certain human rights and freedoms, as they limit freedom of movement and social interactions [6, 39]. Therefore, there is a probability of disregarding or even resisting such rules. When relying on the voluntariness of complying with restrictions and regulations, citizens will follow them only up until they feel that the measures are reasonable. However, as soon as citizens perceive that by abiding measures, they are worse off than their non-complying peers, they will stop complying [40].

General deterrence theory is one of the leading criminological theories, which assumes that people are deterred from breaking the accepted social norms by the certainty, severity, and celerity of punishment [41]. It is one of the deterrence theories, which assume that behavior is a product of rational thinking weighing the costs and benefits of a particular behavior. In this rational process individuals assess their behavior based on the perceived benefits and costs of such actions and subsequently choose the most optimal option [42]. However, studies based on general deterrence theory are varying [42, 43]. Certainty of punishment has been identified as the most influential factor in preventing deviant behavior by most studies [44, 45]. Studies focusing on state-level crime prevention indicate a weak negative correlation between the certainty of punishment and the number of offenses, while severity is only negatively correlated to certain types of crime [46].

Nevertheless, some researchers find that severity is at least as decisive as certainty [44, 47]. The difference in significance of punishment severity and certainty could be explained by the perceived morality of the behavior, which differs among individuals [48]. Individuals with a higher sense of morality tend to be influenced more by the certainty of punishment, while individuals with a lower sense of morality tend to be influenced more by the severity of punishment [47]. Past studies have already distinguished different types of individuals who are differently affected by deterrence. This is mostly dependent on an individual’s sensitivity to threats of sanction [43]. Therefore, it is essential to consider that the prevention of deviant behavior is also influenced by other factors, such as the social environment and personal characteristics of potential perpetrators [49, 50]. Based on these findings, we propose the following hypotheses:

**H2a:** Perceived severity of *formal* punishment is positively associated with compliance with COVID-19 prevention measures.
**H2b:** Perceived certainty of *formal* punishment is positively associated with compliance with COVID-19 prevention measures.

One of the main shortcomings of general deterrence theory is the assumption that the threat of formal punishment acts independently from other dimensions of social surveillance [51]. When we examine the fear of punishment, conformism cannot be attributed solely to formal sanctions. Several studies have also identified informal social surveillance as a critical factor in controlling crime [52]. One study, for example, has shown that more than half of their respondents believe informal surveillance mechanisms are more important than formal ones [53]. Several other studies also pointed out that the threat of informal punishment from parents, friends, and other people, whom individuals perceive as important, has a similar effect on crime prevention as fear of formal punishment [51]. Thus, informal punishment should also be considered in the research of compliance with COVID-19 preventive measures.

To summarize, severity and certainty of punishment have been identified as efficient factors influencing individuals to follow the rules or measures, however mainly in organizational settings [54] and criminology. Hence, complying with protective measures in times of a pandemic has not been researched through the prism of general deterrence theory. Based on these findings, we propose the following hypotheses:

**H2c:** Perceived severity of *informal* punishment is positively associated with compliance with COVID-19 prevention measures.
**H2d:** Perceived certainty of *informal* punishment is positively associated with compliance with COVID-19 prevention measures.

## 3 Method

To answer the research questions and test the hypotheses, we have conducted empirical research. This study focused on cross-sectional data collection during the beginning of the epidemic outbreak, thus when most restrictive protective measures (e.g., prohibition of outdoor movement, temporary restriction on specific work processes) were in force.

At the time of our study, when ordinary and socially accepted behavior (e.g., dining at restaurants, social gatherings, attending classes and seminars, free passing between municipalities) was controlled and monitored, the effect of intimidation was expected to be most significant. The purpose of this study was to identify the factors predicting compliance with prevention measures in specific circumstances when exceptionally restrictive measures, uncertainty, and danger to public health apply. The dependent variable was not based on the reported past behavior or behavioral intention but rather on reporting current behavior. The research was conducted in the form of an online survey among the citizens of Slovenia. By conducting such research, we gained insight into the citizens’ attitudes precisely when new measures resulted in uncertainty on both formal and informal sanctions.

### 3.1 Questionnaire development

To reduce issues with questionnaire reliability and validity, measures used in this study were adopted from previously validated research and adapted to the context of our study. Our research model followed the hypotheses presented in the previous section. Hence, it consists of eight independent variables and one dependent variable. The independent variables derive from the constructs of the Protection motivation theory and the General deterrence theory, while the dependent variable depicts the compliance with COVID-19 prevention measures. Each construct consisted of three items, measured on a five-point Likert scale (ranging from *“strongly disagree”* to *“strongly agree”*), except for attitude on current protective measures, which consisted of seven indicators and was measured using a five-point semantic differential, and severity of the informal punishment which was measured on five-point Likert-type scale from 1 –*Not ashamed at all* to 5 –*Extremely ashamed*. The constructs severity of potential punishment by authorities (i.e., formal severity–FS) and by society (i.e., informal severity–IS) were adapted from [55]. Indicators on the certainty of punishment by authorities (i.e., formal certainty–FC) and by society (informal certainty—IC) were adapted from [56]. Perceived response efficacy (RE) was adapted from [23] and [57], and perceived self-efficacy (SE) was adapted from [23]. Except for one item in each of the following constructs, which were self-developed, the perceived vulnerability to the disease (PV) was adapted from [58] and [59], and the perceived severity (PS) of the disease was adapted from [59]. Items for attitudes on COVID-19 recommended protective behavior were adapted from [60]. Due to the uniqueness of the pandemic situation, items for the dependent variable, compliance with COVID-19 prevention measures (i.e., protective behavior—PB), were self-developed based on the protective measures in force. The questionnaire was initially developed in English and translated to Slovenian by three independent unprofessional translators, experts on self-protection and criminology. The researchers then consolidated the three translations and pre-tested the questionnaire on four respondents who tested the questionnaire for clarity and grammatical soundness. Questionnaire items in Slovene and English with sources are presented in S1 File.

### 3.2 Data collection

Due to the sensitive nature of the research and to protect respondents’ anonymity, the following measures were put in place. First, the survey was created and distributed using a web-based application, which does not allow storing respondents’ IP addresses. Second, respondents were informed about the anonymity of the survey in the introduction. Third, we ensured that the collected data would only be used and stored for research purposes. Additionally, the Commission for Ethics in Research of the University of Maribor, Faculty of Criminal Justice and Security, reviewed the research and issued a positive opinion for conducting the research on April 9, 2020. To reach a diverse sample in the time of restrictions on outdoor movement, we have used a convenience sampling method by distributing the survey to ten groups on the social media network Facebook where Slovenian-speaking users shared their opinions on various topics. Hence, the survey was distributed in groups covering different interest areas (student groups, different interest groups, and groups connected with COVID-19). We aimed to gather a sample size equal to or greater than ten times the number of items forming the constructs of the research model. The survey was active from April 11 until May 2, 2020. We received N = 408 responses, from which eight had to be excluded since they had more than one-third of missing values. Additional two responses were excluded since they included two-thirds of the same values. Hence, our statistical analyses were based on a sample of N = 394 respondents.

Our sample consisted of 76 percent women, while one percent of respondents did not provide an answer to the gender question. The respondent’s ages varied between 15 and 71 years old (M = 31.3; SD = 11.3; Me = 27). Other demographic data of the sample are presented in Table 1.

**Table 1:** Sample demographics.

Characteristic		Number	Share (%)
Education	Less than bachelor’s degree	172	45.7
	Bachelor’s degree	142	37.7
	Master’s degree	58	15.4
	PhD	5	1.3
Status	Student, college student	153	39.3
	Employed, self-employed	199	51.2
	Unemployed	21	5.4
	Retired	10	2.6
Living environment	Rural area	113	29.0
	Town	110	28.3
	Suburbs	50	12.9
	City	116	29.8

## 4 Results

### 4.1 Instrument validation

Before answering the research questions, we have tested the questionnaire for validity and reliability. All statistical analyses in this study were conducted with IBM Statistics SPSS v28. Exploratory factor analysis (*Principal Axis Factoring*) with an orthogonal rotation (*Varimax*) extracted nine theoretically assumed factors with which 72.1 percent of variance can be explained. Kaiser-Meyer-Olkin coefficient (*KMO* = 0.88) indicates meritorious sample adequacy. Bartlett’s test of sphericity was significant (χ^2^ = 7465.05, *df* = 351, *p* < 0.001). The rotated factor matrix with factor loadings is presented in Table 2.

**Table 2:** Rotated factor matrix with factor loadings (loadings lower than .35 are suppressed).

	1	2	3	4	5	6	7	8	9
PB1	-	-	-	-	-	-	-	.676	-
PB2	-	-	-	-	-	-	-	.492	-
PB3	-	-	-	-	-	-	-	.780	-
PV1	-	-	-	-	-	-	-	-	.810
PV2	-	-	-	-	-	-	-	-	.755
PV3	-	-	-	-	-	-	-	-	.475
PS1	-	-	.831	-	-	-	-	-	-
PS2	-	-	.672	-	-	-	-	-	-
PS3	-	-	.717	-	-	-	-	-	-
SE1	-	-	-	-	-	.566	-	-	-
SE2	-	-	-	-	-	.987	-	-	-
SE3	-	-	-	-	-	.779	-	-	-
RE1	-	.787	-	-	-	-	-	-	-
RE2	-	.788	-	-	-	-	-	-	-
RE3	-	.752	-	-	-	-	-	-	-
IS1	.859	-	-	-	-	-	-	-	-
IS2	.883	-	-	-	-	-	-	-	-
IS3	.886	-	-	-	-	-	-	-	-
IC1	-	-	-	-	-	-	.737	-	-
IC2	-	-	-	-	-	-	.819	-	-
IC3	-	-	-	-	-	-	.596	-	-
FS1	-	-	-	-	.685	-	-	-	-
FS2	-	-	-	-	.704	-	-	-	-
FS3	-	-	-	-	.718	-	-	-	-
FC1	-	-	-	.844	-	-	-	-	-
FC2	-	-	-	.618	.434	-	-	-	-
FC3	-	-	-	.802	-	-	-	-	-

Questionnaire reliability was measured with *Cronbach Alpha* (CA) coefficient. Results for individual constructs are situated between α = 0.793 (IC) and α = 0.985 (IS). Other results are presented in Table 3 (diagonally, bold). We calculated inter-construct correlations with the *Pearson* correlation coefficient based on the normality of data distribution and the corresponding CA values (Table 3). Correlation coefficients are below the recommended ideal value (ρ < 0.7), thereby determining the appropriate discriminant validity of the model. Other linear regression assumptions (i.e., multicollinearity, homoscedasticity, normal distribution of residuals) were carefully considered.

**Table 3:** Pearson correlation coefficient and Cronbach alpha (diagonally, bold) *—*p* < .05; **—*p* < .01; ***—*p* < .001.

	FC	SE	IS	IC	RE	PB	FS	PS	PV
1	**.89**								
2	-.01	**.82**							
3	***.35	*-.12	**.99**						
4	***.41	-.03	***.38	**.79**					
5	**.15	**.13	***.43	***.24	**.91**				
6	***.18	-.02	***.53	***.20	***.59	**.83**			
7	***.59	.02	***.51	***.41	***.24	***.34	**.85**		
8	**.16	**-.14	***.47	**.17	***.58	***.63	***.30	**.90**	
9	***.29	***-.29	***.29	***.18	***.31	***.31	***.22	***.45	**.78**

### 4.2 Results of descriptive statistics

To answer **RQ1** and **RQ2**, we have calculated descriptive statistics. In addition to descriptive statistics, a one-sample t-test was performed for all variables with a test value set on mid-point value (3.00). The purpose was to evaluate whether the respondents’ attitudes statistically significantly deviate from the neutral standpoint and in what direction. Results indicate that all constructs significantly deviate from the mid-point value. The constructs certainty of formal punishment (FC) and perceived vulnerability (PV) are the only ones for which the mean is statistically significantly *lower* than the mid-point value, suggesting that respondents did not perceive formal punishment for disobeying mandatory protective measures as certain. Likewise, respondents did not feel vulnerable to COVID-19. Other constructs have statistically significantly *exceeded* the mid-point value. It should be noted that the highest mean and mode are reflected in compliance with protective measures (PB) and their perceived effectiveness (RE). Values of skewness and kurtosis are not exceeding |1.96| indicating an approximately normal data distribution [61]. Results are presented in Table 4 for each construct (mean [M], standard deviation [SD], one-sample t-test [t], median [Me] and mode [Mo]).

**Table 4:** Descriptive statistics results.

	M	SD	*t*	Me	Mo	Skewness	Kurtosis
SE	3.56	.84	***13.20	3.67	4.00	-.09	-.31
RE	3.97	.89	***21.69	4.00	4.00	-.89	.83
PV	2.41	.97	***-12.16	2.33	1.00	.34	-.37
PS	3.70	1.02	***13.51	4.00	4.00	-.70	-.03
PB	4.03	.88	***23.13	4.00	5.00	-.97	.85
IS	3.20	1.37	**2.91	3.00	4.00	-.29	-1.15
IC	3.19	.90	***4.11	3.00	3.00	-.10	-.15
FS	3.16	1.00	**3.22	3.33	3.00	-.26	-.34
FC	2.89	.93	*-2.34	3.00	3.00	.06	-.18

***—*p* < .001

**—*p* < .01

*—*p* < .05.

**Fig 1:**
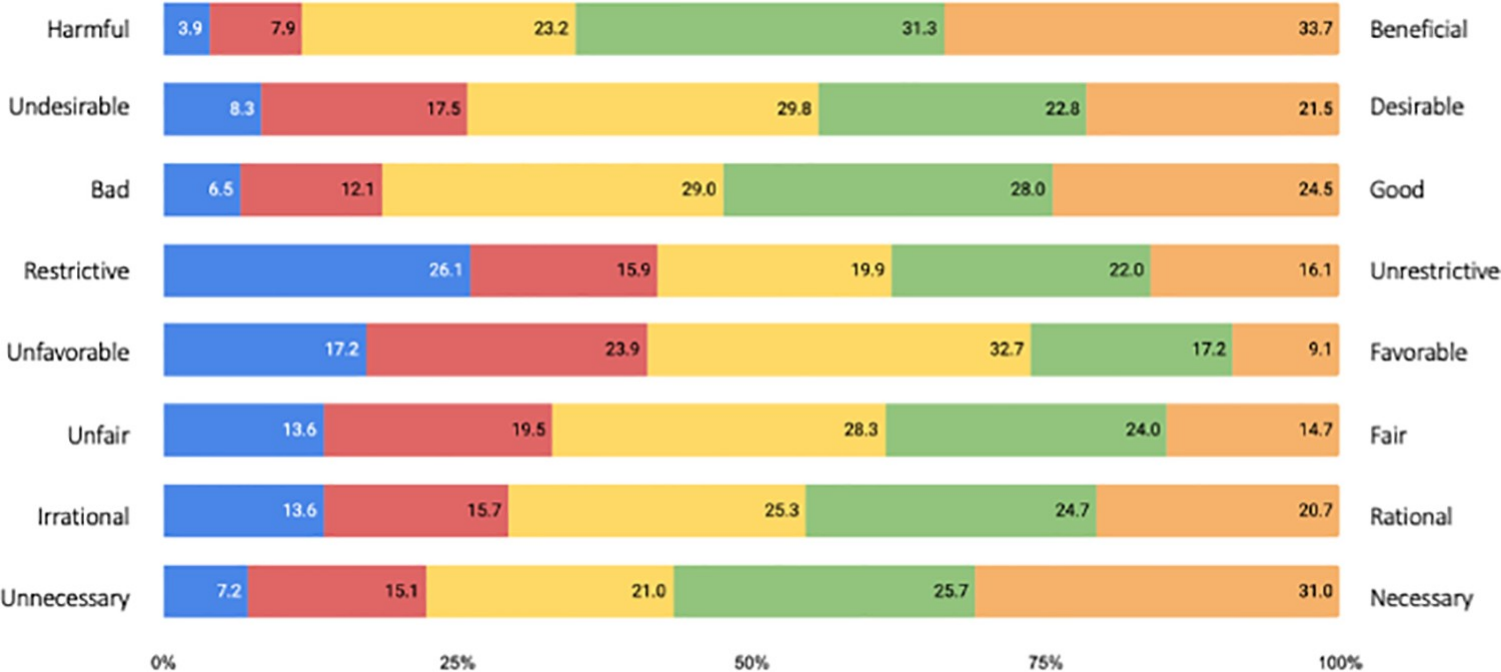
Shares of responses on attitudes toward the protective measures’ directives: *"I think recommended protective instructions given by government and health institutions are*…*"*.

To answer **RQ3**, we have examined the general attitude of respondents to the recommendations of government and experts on self-protective measures. Respondents have provided their responses (on given statements) on a five-point semantic differential scale. The results are presented in Fig 1. The horizontal graph shows the frequency of the selected values as a percentage.

The most frequently chosen value by the respondents, was the mid-point value (*Mo* = 3), with the exception of harmful/beneficial and unnecessary/necessary setting, where the most frequently chosen value was the highest one (*Mo = 5*). The results suggest that the respondents are mostly favorable towards recommended protective measures in the peak of pandemic conditions.

### 4.3 Model test results

**Fig 2:**
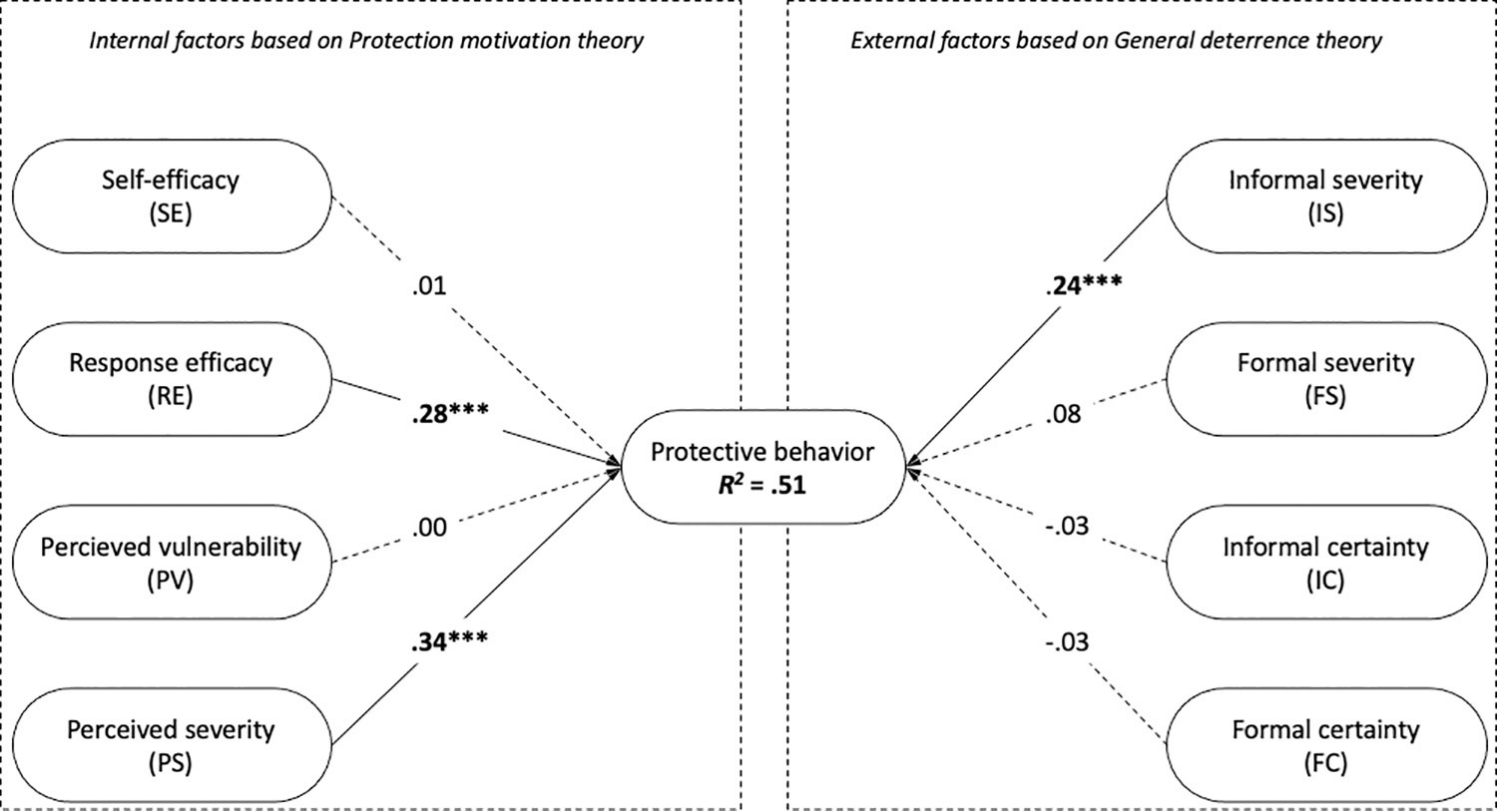
Model test results (*—*p* < .05; ***—*p* < .001).

A multiple linear regression analysis was calculated to predict self-reported protective behavior (PB) based on respondents’ perceptions of formal and informal severity of the punishment (FS, IS), formal and informal certainty of the punishment (FC, IC), their perceived self-efficacy (SE), response efficacy (RE), vulnerability (PV), and severity of the disease (PS), to answer RQ4. A statistically significant regression equation was found (*F*(8, 380) = 50.317, *p* < .001), with *R*^*2*^ = .514 (adjusted *R*^*2*^ = .504). Values of standardized beta coefficients are presented in Fig 2.

Out of all factors included in the analysis, only severity of informal punishment (e.g., social media posts, appearances on television, or other mass media), perceived severity of the infection, and perceived response efficacy were found to be significantly associated with compliance to the COVID-19 recommended preventive behavior. Altogether, 51 percent of variance can be explained by factors included in the research.

## 5 Discussion

This study, conducted among Slovenian-speaking residents of the Republic of Slovenia, resulted in several findings. Data, gathered in our study, refer to the factors that, according to the proposed research model, impact compliance with recommended self-protective measures. It should be considered that the restrictive and repressive protective measures were adopted to combat a novel and specific situation and were thus new to people. As this was a unique situation on a global scale, high levels of uncertainty among the people, stemming from a lot of conflicting information in the media and on social networks, should be considered [62]. At the time of our study, a high level of fear from a new and unknown disease was observed [63] along with spreading misinformation and lack of knowledge [64], which can affect the general support of COVID-19 preventive behavior [65].

Respondents evaluated recommendations on self-protection from government and healthcare experts as beneficial, good and necessary, suggesting that respondents believe that the recommended preventive behavior effectively protects against COVID-19. On the other hand, respondents also believe that the recommendations are unfavorable since, in that short period, the government implemented several measures (based on the recommendations from healthcare experts), which heavily interfered with day-to-day activities and certain personal freedoms. Respondents also believe that the recommendations from government and healthcare experts are more unfair than fair. This can be attributed to the fact that the protective measures were not implemented uniformly across different spheres of society. For example, some business sectors were operating almost normally, whereas all forms of social gatherings (even limited ones) were strictly forbidden.

Among the factors included in our study, self-reported compliance with COVID-19 recommended preventive measures reached the highest mean value. This factor is the only one with its mode reaching the maximum value (*Mo* = 5), suggesting that the respondents, self-reportedly, strictly complied with the recommended preventive measures. However, it should be noted that our research was conducted at the beginning of the pandemic when high levels of uncertainty [62] and fear of the unknown disease [63] reigned among the population. Some studies found, that in the later stages of the pandemics and epidemics, compliance and agreement with preventive measures decrease [66]. The high level of self-reported compliance with COVID-19 preventive measures can also be partially explained by the fact that authorities sanctioned non-compliance and that the respondents consider the measures to be beneficial and necessary, which can positively influence one’s decision to follow the implemented measures [40, 67].

On the other hand, perceived vulnerability to the disease was the factor with the lowest mean value. It is the only factor with its mode reaching the lowest value (*Mo* = 1). This result suggests that respondents believed there is a very low probability of them contracting the COVID-19 disease. Such finding is not in line with most research conducted in the pandemic, as they indicate that perceived vulnerability is associated with protective measures compliance [68–70]. The low mean value of perceived vulnerability can be partially explained by the fact that the majority of the respondents in our sample were not part of a critical age group and thus felt less threatened by the disease.

Furthermore, our research results have shown that the severity of punishment affects the decision to comply with preventive measures more than the certainty of punishment in pandemic situations. Even though previous research suggested the certainty of punishment is more important than the severity of punishment in preventing deviant behavior, several other studies also pointed out the importance of the severity of punishment [47]. Studies suggesting that the severity of punishment is a more important predictor than certainty usually attribute the difference to different levels of the morality of respondents, with the majority of those studies focusing on workplace deviance [71]. In contrast, our study focuses on compliance with COVID-19 preventive measures in times of the pandemic, which has not yet been investigated. The finding that respondents in this novel situation attribute more importance to the severity of punishment can be explained by the fact that the certainty of punishment is heavily influenced by experience [72]. Since the COVID-19 situation and the protective measures are novel, people do not have any experience with the certainty of punishment in terms of non-compliance with mandatory preventive measures. However, on the other hand, the severity of punishment is heavily influenced by public exposure of punishment and emphasizing norm violations [47]. Both could be readily and frequently observed in Slovenian media from the pandemic’s beginning [11]. Such results are also in line with other studies, which explored the effect of deterrence on different groups of people. The severity of punishment has been identified as a significant factor for conformists and deterrable offenders, whereby the majority of the population can be classified in the latter group. These groups also attach more importance to informal surveillance than those who cannot be influenced by deterrence [43].

Furthermore, our results indicate that the fear of informal punishment could be a more important predictor of compliance with COVID-19 preventive measures than the threat of formal punishment. Informal punishment, which has already been identified as more important than formal punishment by several other studies [53, 55], is more likely to occur (and be effective in preventing violations of norms) when the community supports such norms and considers them necessary [73]. Hence, the respondents’ relatively positive attitude towards COVID-19 preventive measures may also explain the significance of informal punishment. Such results can be attributed to the fact that people in similar emergencies are emotionally charged, uncertain, and seek a sense of comfort, justice, or even entertainment in public shaming over social networks [8, 74]. Additionally, non-compliance with COVID-19 preventive measures was relatively easy for the public to spot with informal social surveillance, as the protective measures were primarily applied in public.

Among the PMT factors included in our research, the disease’s response efficacy and perceived severity were statistically significant. Contrary to the factors derived from general deterrence theory, both factors stem from an individual’s thoughts and beliefs [16]. Statistical significance of response efficacy can be partially attributed to the positive attitude of the respondents towards the protective measures, while the statistical significance of perceived severity of infection can partially be attributed to the awareness about the disease. Even though respondents are not worried about contracting the virus, they comply with COVID-19 preventive measures to protect vulnerable social groups. Another study conducted at the time of the COVID-19 pandemic came to a similar conclusion [75]. These results are in line with other studies, which suggest that compliance with accepted norms can not only be attributed to the threat of deterrence but must also be attributed to personal differences and beliefs [49]. Furthermore, the finding that the perceived severity of infection and response efficacy are both significant predictors of compliance with COVID-19 preventive measures is also in line with other studies, which investigated PMT factors in times of pandemics [14, 76, 77].

The presented research results significantly complement other studies, addressing behavior and compliance with preventive measures in times of epidemics and pandemics. By investigating the factors derived from general deterrence theory, we advance and progress the findings of previous studies, which primarily focused on factors in the domain of an individual–internal factors [14, 76]. Along with formal punishment, which is usually associated with general deterrence theory, our results have additionally pointed out the importance of informal punishment in times of the pandemic. Our study also supplements findings from previous studies, which focused on general deterrence theory, as the results showed that severity of punishment is a more important predicting factor than the certainty of punishment in times of a pandemic or other emergency. In the past, such conclusions were mainly associated with workplace deviance [71] and traffic safety [56].

### 5.1 Research implications

Results of our research offer several theoretical as well as practical implications. First, the pioneering use of general deterrence theory in times of a pandemic can be counted as one of the most important theoretical implications. Previous studies have tried to explain human behavior using broader theories, such as the theory of planned behavior, the protection motivation theory, and the health belief model. Although previously mentioned theories provide an appropriate and reasonable explanation for human behavior, they still neglect extraordinary and restrictive governmental interventions in the residents’ daily lives. This study is thus the first one to offer an empirical insight into compliance with COVID-19 preventive measures and attitudes on these measures during COVID-19 by using the general deterrence theory. Second, the study was conducted when Slovenia declared the epidemic and various mandatory preventive measures were in force. In addition to this research offering insights through the lens of the protection motivation theory and the general deterrence theory, it also offers a direct insight into citizens’ attitudes and behavior in times of the most restrictive measures and general uncertainties. Third, this study allows for an understanding of the effectiveness of certainty and severity of formal and informal punishment. Our results suggest that informal punishment, and especially the informal severity has the most significant effect on compliance with COVID-19 protective measures within the factors of general deterrence theory. Such (relatively unconventional) results may be attributed to the high levels of uncertainty and lack of experience with formal punishments of protective measures violations in times of pandemic. Deterrence stemming from informal social control was spread daily from different media sources. Hence, it appears it impacted citizens’ behavior more than deterrence from the government. Further research in general deterrence theory should thus also include informal deterrence actors as essential factors influencing compliance with accepted norms. Fourth, to the best of our knowledge, we are the first to include internal factors from the protection motivation theory into our research model, based on the general deterrence theory. The results show that the factors from the protection motivation theory more significantly impact citizens’ compliance to COVID-19 protective measures than factors deriving from the general deterrence theory. Such a result suggests it is essential to explore internal factors when investigating factors associated with compliance to mandatory protective measures.

In times of such emergency as the COVID-19 pandemic, recommendations from healthcare experts, requirements by the government, and media reporting should be unified. The extreme levels of uncertainty about the current situation and future outcomes may result in fear for existence. As our results show, people seem to attach great importance to the responses of the social environment in such situations. Therefore, media reporting should be done responsibly and following formal surveillance guidelines. Otherwise, media coverage can increase (unreasonable) fear and uncertainty in public or even create doubt in the viability of formal punishment. Our study also suggests that certainty should also be emphasized in addition to emphasizing the severity of punishment. The novel measures also bring uncertainty in the consistency of legal execution. Hence, perceiving punishment as certain can be strengthened by consistent and fair enforcement of the law. Furthermore, this research also shows the need to address the internal factors, such as the perceived severity of the infection and response efficacy. Based on our results, both factors have a more significant impact on an individual’s decision to comply with the COVID-19 preventive measures than any form of deterrence. Hence, it is critical to provide the public with appropriate means to follow the COVID-19 preventive measures, clear instructions on carrying out the protective measures, and most importantly, sufficient and unbiased explanation on why it is essential to follow the measures.

### 5.2 Limitations and future work

While interpreting the results of our study, several limitations need to be considered. First, the survey was only shared on the Facebook social network among Slovenian-speaking users. Given the conditions in which the research was conducted, field surveys were not possible. The diversity of the sample was ensured by sharing the survey in different interest groups inside Facebook. Second, compliance with the COVID-19 preventive measures was self-reported, leaving a possibility of biased answers. Third, our sample mainly consisted of women respondents, which may show higher levels of conformism and are more influenced by informal surveillance than men [53].

Following the limitations mentioned above, this study should be repeated to observe the changes longitudinally. Such a research design would enable monitoring of attitudes and self-reported behavior changes over time, and possibly reach a more representative sample. In unpredictable circumstances, individuals tend to respond inconsistently, and their attitudes may change more quickly than in circumstances with which they are familiar. Furthermore, it would be reasonable to create additional indicators for the measured constructs to measure the attitudes and behavior from different viewpoints and capture a broader picture of variables.

## Supporting information

S1 File Questionnaire items. Questionnaire items in original and English language with references.
(DOCX)

## References

[1] Toelstede B. Democracy interrupted: The anti-social side of intensified policing. Democr Secur. 2019;15: 137–149. doi: 10.1080/17419166.2018.1493992

[2] Gostin LO. When terrorism threatens health: How far are limitations on human rights justified. J Law, Med Ethics. 2003;31: 524–528. 14968655 10.1111/j.1748-720x.2003.tb00120.x

[3] Weston J. Sites of sickness, sites of rights? HIV / AIDS and the limits of human rights in British prisons. Cult Soc Hist. 2019;16: 225–240. doi: 10.1080/14780038.2019.1585012 31708693 PMC6817313

[4] Wilson K, Brownstein JS, Fidler DP. Strengthening the International Health Regulations: lessons from the H1N1 pandemic. Health Policy Plan. 2010;25: 505–509. doi: 10.1093/heapol/czq026 20595331 PMC2957909

[5] Frieden TR, Damon I, Bell BP, Kenyon T, Nichol S. Ebola 2014—New Challenges, New Global Response and Responsibility. N Engl J Med. 2014;371: 1177–1180. doi: 10.1056/NEJMp1409903 25140858

[6] Balachandar V, Mahalaxmi I, Kaavya J, Vivekanandhan G, Ajithkumar S, Arul N, et al. COVID-19: Emerging protective measures. Eur Rev Med Pharmacol Sci. 2020;24: 3422–3425. doi: 10.26355/eurrev_202003_20713 32271461

[7] Siedner MJ, Harling G, Reynolds Z, Gilbert RF, Haneuse S, Venkataramani AS, et al. Social distancing to slow the US COVID-19 epidemic: Longitudinal pretest–posttest comparison group study. PLoS Med. 2020;17. doi: 10.1371/JOURNAL.PMED.1003244 32780772 PMC7418951

[8] Kohm SA. Naming, shaming and criminal justice: Mass-mediated humiliation as entertainment and punishment. Crime, Media, Cult. 2009;5: 188–205. doi: 10.1177/1741659009335724

[9] Hart PS, Chinn S, Soroka S. Politicization and Polarization in COVID-19 News Coverage. Sci Commun. 2020;42: 679–697. doi: 10.1177/1075547020950735 PMC7447862 38602988

[10] Prosser AMB, Judge M, Bolderdijk JW, Blackwood L, Kurz T. ‘Distancers’ and ‘non-distancers’? The potential social psychological impact of moralizing COVID-19 mitigating practices on sustained behaviour change. Br J Soc Psychol. 2020;59: 653–662. doi: 10.1111/bjso.12399 32584437 PMC7361762

[11] Šuligoj B. Nova ponudba portoroškega turizma. In: Delo [Internet]. 2020 [cited 5 May 2020]. Available: https://www.delo.si/novice/slovenija/nova-ponudba-portoroskega-turizma-305053.html.

[12] Ahmed MZ, Ahmed O, Aibao Z, Hanbin S, Siyu L, Ahmad A. Epidemic of COVID-19 in China and associated psychological problems. Asian J Psychiatr. 2020;51. doi: 10.1016/j.ajp.2020.102092 32315963 PMC7194662

[13] Yan QL, Tang SY, Xiao YN. Impact of individual behaviour change on the spread of emerging infectious diseases. Stat Med. 2018;37: 948–969. doi: 10.1002/sim.7548 29193194

[14] Williams L, Rasmussen S, Kleczkowski A, Maharaj S, Cairns N. Protection motivation theory and social distancing behaviour in response to a simulated infectious disease epidemic. Psychol Heal Med. 2015;20: 832–837. doi: 10.1080/13548506.2015.1028946 25835044

[15] Zhang X, Wang F, Zhu C, Wang Z. Willingness to self-isolate when facing a pandemic risk: Model, empirical test, and policy recommendations. Int J Environ Res Public Health. 2020;17. doi: 10.3390/ijerph17010197 31892171 PMC6981847

[16] Bish A, Michie S. Demographic and attitudinal determinants of protective behaviours during a pandemic: A review. Br J Health Psychol. 2010;15: 797–824. doi: 10.1348/135910710X485826 20109274 PMC7185452

[17] Machida M, Nakamura I, Saito R, Nakaya T, Hanibuchi T, Takamiya T, et al. Adoption of personal protective measures by ordinary citizens during the COVID-19 outbreak in Japan. Int J Infect Dis. 2020. doi: 10.1016/j.ijid.2020.04.014 32283285 PMC7194542

[18] Zhang CQ, Chung PK, Liu JD, Chan DKC, Hagger MS, Hamilton K. Health Beliefs of Wearing Facemasks for Influenza A/H1N1 Prevention: A Qualitative Investigation of Hong Kong Older Adults. Asia-Pacific J Public Heal. 2019;31: 246–256. doi: 10.1177/1010539519844082 31007032

[19] Wong CY, Tang CSK. Practice of habitual and volitional health behaviors to prevent severe acute respiratory syndrome among Chinese adolescents in Hong Kong. J Adolesc Heal. 2005;36: 193–200. doi: 10.1016/j.jadohealth.2004.02.024 15737774 PMC7129542

[20] Yang ZJ. Predicting young adults intentions to get the H1N1 vaccine: An integrated model. J Health Commun. 2015;20: 69–79. doi: 10.1080/10810730.2014.904023 24870976

[21] Rogers RW. A Protection Motivation Theory of Fear Appeals and Attitude Change. J Psychol. 1975;91: 93–114. doi: 10.1080/00223980.1975.9915803 28136248

[22] Rosenstock IM. Historical Origins of the Health Belief Model. Health Educ Monogr. 1974;2: 328–335.

[23] Milne S, Orbell S, Sheeran P. Combining motivational and volitional interventions to promote exercise participation: Protection motivation theory and implementation intentions. Br J Health Psychol. 2002;7: 163–184. doi: 10.1348/135910702169420 14596707

[24] Rogers RW. Cognitive and physiological process in fear appeals and attitude change: a revised theory of protection motivation. In: Cacioppo J, Petty R, editors. Social Psychophysiology: a source book. New York: Guilford Press; 1983. pp. 153–176.

[25] Boer H, Seydel ER. Protection motivation theory. In: Connor M, Norman P, editors. Predicting Health Behavior. Buckingham: Open University Press; 1996. pp. 95–120. doi: 10.1080/13548506.2011.579983

[26] Flynn MF, Lyman RD, Prentice-Dunn S. Protection motivation theory and adherence to medical treatment regimens for muscular dystrophy. J Soc Clin Psychol. 1995;14: 61–75.

[27] LeFebvre R. The human element in cyber security: A study on student motivation to act. Proceedings of the 2012 Information Security Curriculum Development Conference, InfoSec CD 2012. New York: ACM; 2012. pp. 1–8. doi: 10.1145/2390317.2390318

[28] Neuwirth K, Dunwoody S, Griffin R. Protection Motivation and Risk Communication. Risk Anal. 2000;20: 721–734. doi: 10.1111/0272-4332.205065 11110218

[29] Mihelič A, Vrhovec S. Explaining the employment of information security measures by individuals in organizations: The self-protection model. In: Bernik I, Markelj B, Vrhovec S, editors. Advances in Cybersecurity. Maribor: University of Maribor Press; 2017.

[30] Jansen J. Studying Safe Online Banking Behaviour: A Protection Motivation Theory Approach. Proc Ninth Int Symp Hum Asp Inf Secur Assur (HAISA 2015) Stud. 2015; 120–130.

[31] Mihelič A. Samovarovanje pred spletnimi napadi v organizacijah. Univerza v Mariboru. 2017.

[32] Martens M, Wolf R De, Marez L De. Computers in Human Behavior Investigating and comparing the predictors of the intention towards taking security measures against malware, scams and cybercrime in general. Comput Human Behav. 2019;92: 139–150. doi: 10.1016/j.chb.2018.11.002

[33] Maddux JE, Rogers RW. Protection motivation theory and self-efficacy: A revised theory of fear appeals and attitude change. J Exp Soc Psychol. 1983;19: 469–479.

[34] Lazarus R, Folkman S. Stress, coping, and adaptation. New York: Springer-Verlag; 1984.

[35] Boss SR, Street F, Galletta DF, Lowry PB, Kong H, Moody GD, et al. What do systems users have to fear? Using fear appeals to engender threaths and fear that motivate protective security behaviors. MIS Q. 2015;39: 1–13.

[36] Johnston BAC, Warkentin M. Fear appeals and information security behaviors: An empirical study. MIS Q. 2010;34: 549–566.

[37] Savage M. Coronavirus: Has Sweden got its science right? In: BBC News [Internet]. 2020 [cited 28 Apr 2020]. Available: https://www.bbc.com/news/world-europe-52395866.

[38] Government of the Republic of Serbia. Government adopts new measures as COVID-19 response. 2020 [cited 28 Apr 2020]. Available: https://www.srbija.gov.rs/vest/en/152070/government-adopts-new-measures-as-covid-19-response.php.

[39] Teslya A, Pham TM, Godijk NG, Kretzschmar ME, Bootsma MCJ, Rozhnova G. Impact of self-imposed prevention measures and short-term government-imposed social distancing on mitigating and delaying a COVID-19 epidemic: A modelling study. PLoS Med. 2020;17: 1–21. doi: 10.1371/journal.pmed.1003166 32692736 PMC7373263

[40] Ambrož M. Namen kaznovanja: pozitivna generalna prevencija? Rev za Kriminalistiko Kriminologijo. 2016;67: 5–15.

[41] Gibbs JP. Crime, Punishment, and Deterrence. Southwest Soc Sci Q. 1968;48: 515–530.

[42] Meško G, Hirtenlehner H, Bertok E. Vloga samonadzora in zastrasevanja v situacijskoakcijski teoriji (SAT)—rezultati slovenske študije v srednjih šolah. Rev za Kriminalistiko Kriminologijo. 2015;66: 33–43.

[43] Pogarsky G. Identifying “deterrable” offenders: Implications for research on deterrence. Justice Q. 2002;19: 431–452. doi: 10.1080/07418820200095301

[44] Nagin DS, Pogarsky G. Integrating Celerity, Impulsivity, and Extralegal Sanction Threats Into a Model of General Deterrence: Theory and Evidence. Criminology. 2001;39: 865–892. doi: 10.1111/j.1745-9125.2001.tb00943.x

[45] Hirsch A von Bottoms AE, Burney E Wilkstrom PO. Criminal Deterrence and Sentencing Severity. Cambridge, United Kingdom: Hart; 1999.

[46] Silberman M. Toward a Theory of Criminal Deterrence. Am Sociol Rev. 1976;41: 442. doi: 10.2307/2094253

[47] D’Arcy J, Hovav A, Galletta D. User awareness of security countermeasures and its impact on information systems misuse: A deterrence approach. Inf Syst Res. 2009;20: 79–98. doi: 10.1287/isre.1070.0160

[48] MacCoun RJ. Drugs and the law: A psychological analysis of drug prohibition. Psychol Bull. 1993;113: 497–512. doi: 10.1037/0033-2909.113.3.497 8316611

[49] Sherman LW. Defiance, deterrence, and irrelevance: A theory of the criminal sanction. J Res Crime Delinq. 1993;30: 445–473. doi: 10.1177/0022427893030004006

[50] Hirtenlehner H, Pauwels LJR, Meško G. Is the effect of perceived deterrence on juvenile offending contingent on the level of self-control? Results from three countries. Br J Criminol. 2013;54: 128–150. doi: 10.1093/bjc/azt053

[51] Bishop DM. Legal and extralegal barriers to delinquency. Criminology. 1984;22: 403–419.

[52] Vélez ME. Informal Social Control. In: Bruinsma G, Weisburd D, editors. Encyclopedia of Criminology and Criminal Justice. New York: Springer Science+Business Media; 2014. doi: 10.1007/978-1-4614-5690-2

[53] Lambert EG, Jaishankar K, Jiang S, Pasupuleti S, Bhimarasetty J V. Correlates of Formal and Informal Social Control on Crime Prevention: An Exploratory Study among University Students, Andhra Pradesh, India. Asian J Criminol. 2012;7: 239–250. doi: 10.1007/s11417-011-9108-9

[54] Kuo KM, Talley PC, Hung MC, Chen YL. A Deterrence Approach to Regulate Nurses’ Compliance with Electronic Medical Records Privacy Policy. J Med Syst. 2017;41: 1–10. doi: 10.1007/s10916-016-0650-y 29098428

[55] Moody GD, Siponen M, Pahnila S. Toward a unified model of information security policy compliance. MIS Q. 2018;42: 285–311. doi: 10.25300/MISQ/2018/13853

[56] Allen S, Murphy K, Bates L. What drives compliance? The effect of deterrence and shame emotions on young drivers’ compliance with road laws. Polic Soc. 2017;27: 884–898. doi: 10.1080/10439463.2015.1115502

[57] Thrasher JF, Swayampakala K, Borland R, Nagelhout G, Yong HH, Hammond D, et al. Influences of Self-Efficacy, Response Efficacy, and Reactance on Responses to Cigarette Health Warnings: A Longitudinal Study of Adult Smokers in Australia and Canada. Health Commun. 2016;31: 1517–1526. doi: 10.1080/10410236.2015.1089456 27135826 PMC4972657

[58] Ho HS. Use of face masks in a primary care outpatient setting in Hong Kong: Knowledge, attitudes and practices. Public Health. 2012;126: 1001–1006. doi: 10.1016/j.puhe.2012.09.010 23153561 PMC7111693

[59] Myers LB, Goodwin R. Determinants of adults’ intention to vaccinate against pandemic swine flu. BMC Public Health. 2011;11: 1–8. doi: 10.1186/1471-2458-11-1 21211000 PMC3024930

[60] Zhang Y, Yang H, Cheng P, Luqman A. Predicting consumers’ intention to consume poultry during an H7N9 emergency: an extension of the theory of planned behavior model. Hum Ecol Risk Assess. 2020;26: 190–211. doi: 10.1080/10807039.2018.1503931

[61] Darren G, Mallery P. SPSS for Windows Step by Step: A Simple Guide and Reference. Boston, Massachusetts: Allyn & Bacon; 2002.

[62] Ölcer S, Yilmaz-Aslan Y, Brzoska P. Lay perspectives on social distancing and other official recommendations and regulations in the time of COVID-19: A qualitative study of social media posts. BMC Public Health. 2020;20: 1–9. doi: 10.1186/s12889-019-7969-5 32560716 PMC7303937

[63] Fofana NK, Latif F, Bashir MF, Komal B. Fear and Agony of the Pandemic Leading to Stress and mental illness: An Emerging Crisis in the Novel Coronavirus (COVID-19) Outbreak. Psychiatry Res. 2020; 10. doi: 10.1016/j.psychres.2020.113230 32593067 PMC7833263

[64] Li ZH, Zhang XR, Zhong WF, Song WQ, Wang ZH, Chen Q, et al. Knowledge, attitudes, and practices related to coronavirus disease 2019 during the outbreak among workers in China: A large cross-sectional study. PLoS Negl Trop Dis. 2020;14. doi: 10.1371/journal.pntd.0008584 32941447 PMC7498029

[65] Witte K, Allen M. A Meta-Analysis of Fear Appeals: Implications for Effective Public Health Campaigns. Heal Educ Behav. 2000;27: 591–615. doi: 10.1177/109019810002700506 11009129

[66] De Coninck D, D’Haenens L, Matthijs K. Perceptions and opinions on the COVID-19 pandemic in Flanders, Belgium: Data from a three-wave longitudinal study. Data Br. 2020;32. doi: 10.1016/j.dib.2020.106060 32766413 PMC7376332

[67] Tang KHD. Movement control as an effective measure against Covid-19 spread in Malaysia: an overview. J Public Heal. 2020; 17–20. doi: 10.1007/s10389-020-01316-w 32837842 PMC7293423

[68] Ezati Rad R, Mohseni S, Kamalzadeh Takhti H, Hassani Azad M, Shahabi N, Aghamolaei T, et al. Application of the protection motivation theory for predicting COVID-19 preventive behaviors in Hormozgan, Iran: a cross-sectional study. BMC Public Health. 2021;21: 466. doi: 10.1186/s12889-021-10500-w 33685426 PMC7938277

[69] Prasetyo YT, Castillo AM, Salonga LJ, Sia JA, Seneta JA. Factors affecting perceived effectiveness of COVID-19 prevention measures among Filipinos during Enhanced Community Quarantine in Luzon, Philippines: Integrating Protection Motivation Theory and extended Theory of Planned Behavior. Int J Infect Dis. 2020;99: 312–323. doi: 10.1016/j.ijid.2020.07.074 32768695 PMC7406473

[70] Al-Rasheed M. Protective Behavior against COVID-19 among the Public in Kuwait: An Examination of the Protection Motivation Theory, Trust in Government, and Sociodemographic Factors. Soc Work Public Health. 2020;35: 546–556. doi: 10.1080/19371918.2020.1806171 32970542

[71] Vance A, Siponen M. IS security policy violations: A rational choice perspective. J Organ End User Comput. 2012;24: 21–41. doi: 10.4018/joeuc.2012010102

[72] Wenzel M. The social side of sanctions: Personal and social norms as moderators of deterrence. Law Hum Behav. 2004;28: 547–567. doi: 10.1023/b:lahu.0000046433.57588.71 15638209

[73] Petee TA, Milner TF, Welch MR. Levels of Social Integration in Group Contexts and the Effects of Informal Sanction Threat on Deviance. Criminology. 1994;32: 85–106. doi: 10.1111/j.1745-9125.1994.tb01147.x

[74] Heo M, Park J. Shame and vicarious shame in the news: A case study of the Sewol ferry disaster. Journalism. 2019;20: 1611–1629. doi: 10.1177/1464884916688928

[75] Okuhara T, Okada H, Kiuchi T. Predictors of staying at home during the covid-19 pandemic and social lockdown based on protection motivation theory: A cross-sectional study in japan. Healthc. 2020;8. doi: 10.3390/healthcare8040475 33187368 PMC7712029

[76] Miller S, Yardley L, Little P. Development of an intervention to reduce transmission of respiratory infections and pandemic flu: Measuring and predicting hand-washing intentions. Psychol Heal Med. 2012;17: 59–81. doi: 10.1080/13548506.2011.564188 21644184

[77] Mortada E, Abdel-Azeem A, Al Showair A, Zalat MM. Preventive behaviors towards covid-19 pandemic among healthcare providers in Saudi Arabia using the protection motivation theory. Risk Manag Healthc Policy. 2021;14: 685–694. doi: 10.2147/RMHP.S289837 33628067 PMC7898786

