# Supplementary material for: Internal and external drivers for compliance with the COVID-19 preventive measures in Slovenia: The view from general deterrence and protection motivation
Source: PLoS One. 2021 Nov 15;16(11):e0259675. doi: 10.1371/journal.pone.0259675 (PMC8592422; doi:10.1371/journal.pone.0259675)
Supplement: S1 File — Questionnaire items in original and English language with references. (DOCX) [file pone.0259675.s001.docx]

| **S1 File. Questionnaire items.** | | |  |  |
| --- | --- | --- | --- | --- |
| **Construct** | **Code** | **Item in Slovene** | **Item in English** | **Adapted from** |
| Attitude | A1 | Menim, da so priporočena zaščitna navodila, dana s strani vlade in zdravstvenih ustanov:  škodljiva - - - - - koristna. | I think recommended protective instructions given by government and health institutions are: harmful - - - - - beneficial. | [18] |
|  | A2 | Menim, da so priporočena zaščitna navodila, dana s strani vlade in zdravstvenih ustanov:  nezaželena - - - - - zaželena. | I think recommended protective instructions given by government and health institutions are: undesirable - - - - - desirable. | [18] |
|  | A3 | Menim, da so priporočena zaščitna navodila, dana s strani vlade in zdravstvenih ustanov:  slaba -­­ - - - - dobra. | I think recommended protective instructions given by government and health institutions are: bad - - - - - good. | [18] |
|  | A4 | Menim, da so priporočena zaščitna navodila, dana s strani vlade in zdravstvenih ustanov:  omejevalna -­­ - - - - neomejevalna. | I think recommended protective instructions given by government and health institutions are: restrictive - - - - - unrestrictive. | [18] |
|  | A5 | Menim, da so priporočena zaščitna navodila, dana s strani vlade in zdravstvenih ustanov:  neugodna -­­ - - - - ugodna. | I think recommended protective instructions given by government and health institutions are: unfavorable - - - - - favorable. | [18] |
|  | A6 | Menim, da so priporočena zaščitna navodila, dana s strani vlade in zdravstvenih ustanov:  nepravična -­­ - - - - pravična. | I think recommended protective instructions given by government and health institutions are: unfair - - - - - fair. | [18] |
|  | A7 | Menim, da so priporočena zaščitna navodila, dana s strani vlade in zdravstvenih ustanov:  nerazumna -­­ - - - - razumna. | I think recommended protective instructions given by government and health institutions are: irrational - - - - - rational. | [18] |
|  | A8 | Menim, da so priporočena zaščitna navodila, dana s strani vlade in zdravstvenih ustanov:  nepotrebna -­­ - - - - potrebna. | I think recommended protective instructions given by government and health institutions are: unnecessary - - - - - necessary. | [18] |
| Protective behavior | PB1 | Sledim navodilom o samozaščitnih ukrepih, ki jih je podala medicinska stroka. | I am following the instructions regarding self-protective actions given by medical professionals. | self-developed |
|  | PB2 | Sledim navodilom o samozaščitnih ukrepih, ki jih je podala vlada. | I am following the instructions regarding self-protective actions given by the authorities. | self-developed |
|  | PB3 | Sledim navodilom o samozaščitnih ukrepih, ki so jih podale mednarodne medicinske in zdravstvene organizacije. | I am following the instructions regarding self-protective actions given by international medical and health organisations. | self-developed |
| Perceived vulnerability | PV1 | Obstaja visoka možnost, da se bom v naslednjih tednih okužil s COVID-19. | There is a high chance of me getting COVID-19. | [58] |
|  | PV2 | Možnost okužbe s COVID-19 je zame zelo realna. | Getting COVID-19 is currently a high possibility for me. | [59] |
|  | PV3 | Počutim se ranljivega za COVID-19. | I feel vulnerable to COVID-19. | self-developed |
| Perceived severity | PS1 | Zdravstveni zapleti povezani s COVID-19 so resni. | Complications from COVID-19 are serious. | [59] |
|  | PS2 | Če oseba dobi COVID-19, lahko resno zboli. | If a person gets a COVID-19, he/she could get very sick. | [59] |
|  | PS3 | COVID-19 je nevarna bolezen. | COVID-19 is a dangerous disease. | self-developed |
| Self-efficacy | SE1 | Prepričan sem v svojo sposobnost zaščite pred COVID-19. | I feel confident in my ability to protect myself from COVID-19. | [23] |
|  | SE2 | Ni se težko zaščititi pred COVID-19. | It is not difficult to protect myself from COVID-19. | [23] |
|  | SE3 | Samozaščita pred virusom COVID-19 je preprosta. | Protecting myself from COVID-19 is easy for me. | [23] |
| Response efficacy | RE1 | Samozaščitni ukrepi so dober način za zmanjšanje tveganja, da dobim COVID-19. | Self-protective actions are a good way of reducing the risk of me getting COVID-19. | [23] |
|  | RE2 | Če bi deloval samozaščitno, bi zmanjšal tveganje, da dobim COVID-19. | If I were to take self-protective actions, my chances of getting COVID-19 would be lower. | [23] |
|  | RE3 | Samozaščitni ukrepi so učinkovit način za zmanjšanje moje ranljivosti za okužbo s COVID-19. | The self-protective actions are an efficient way of reducing my vulnerability to COVID-19. | [57] |
| Informal severity | IS1 | Kako sram bi vas bilo, če bi vas ujeli med kršenjem zaščitnih ukrepov/navodil in bi se pojavili na družbenih omrežjih. | How ashamed would you be if you were to be caught violating the COVID-19 protective instructions and appear on social networks. | [55] |
|  | IS2 | Kako sram bi vas bilo, če bi vas ujeli med kršenjem zaščitnih ukrepov/navodil in bi se pojavili na televiziji. | How ashamed would you be if you were to be caught violating the COVID-19 protective instructions and appear on TV. | [55] |
|  | IS3 | Kako sram bi vas bilo, če bi vas ujeli med kršenjem zaščitnih ukrepov/navodil in bi se pojavili v drugih množičnih medijih. | How ashamed would you be if you were to be caught violating the COVID-19 protective instructions and appear on other mass media (e.g., newspapers, radio, news websites). | [55] |
| Informal certainty | IC1 | Drugi ljudje bi me zelo verjetno zasramovali, če bi kršil zaščitna navodila. | Other people are very likely to shame me if I would violate protective instructions. | [78] |
|  | IC2 | Zelo verjetno je, da bi me drugi ljudje obsojali, če bi kršil zaščitna navodila. | There is a high possibility that I would be condemned by other people, if I would violate protective instructions. | [78] |
|  | IC3 | Če bi kršil zaščitna navodila, obstaja velika verjetnost, da bi me drugi ljudje prijavili oblastem. | If I would violate protective instructions there are high odds that I would be reported to the authorities by other people. | [78] |
| Formal severity | FS1 | Če bi me oblasti zasačile pri kršenju navodil za zaščito pred virusom COVID-19 bi bil to zame velik problem. | If i were to be caught by the authorities violating the COVID-19 protective instructions it would be a serious problem for me. | [55] |
|  | FS2 | Če bi me oblasti zasačile pri kršenju navodil za zaščito pred virusom COVID-19 bi bil deležen stroge kazni. | If i were to be caught by the authorities violating the COVID-19 protective instructions I would receive a severe penalty. | [55] |
|  | FS3 | Če bi me oblasti zasačile pri kršenju navodil za zaščito pred virusom COVID-19 bi mi to otežilo življenje. | If i were to be caught by the authorities violating the COVID-19 protective instructions It would make my life difficult. | [55] |
| Formal certainty | FC1 | Če bi prekršil navodila za zaščito pred COVID-19 obstaja velika verjetnost, da bi me organi pregona zaznali. | If I were to violate the COVID-19 protective instructions there are high odds that I would be detected by the authorities. | [78] |
|  | FC2 | Če bi prekršil navodila za zaščito pred COVID-19 bi me organi pregona zelo verjetno kaznovali. | If I were to violate the COVID-19 protective instructions I am very likely to be punished by the authorities. | [78] |
|  | FC3 | Če bi prekršil navodila za zaščito pred COVID-19 bi me organi pregona zelo verjetno odkrili. | If I were to violate the COVID-19 protective instructions it is certain that I would be caught by the authorities. | [78] |
